# First person – Jenny Vermeer and Jonathan lent

**DOI:** 10.1242/dmm.046292

**Published:** 2020-07-30

## Abstract

First Person is a series of interviews with the first authors of a selection of papers published in Disease Models & Mechanisms, helping early-career researchers promote themselves alongside their papers. Jenny Vermeer and Jonathan lent are co-first authors on ‘A lineage-tracing tool to map the fate of hypoxic tumour cells’, published in DMM. Jenny conducted the research described in this article while a postdoctoral researcher in the lab of Ruth Muschel at the University of Oxford, Oxford, UK. She is now a project leader in the lab of Miranda van der Lee at Byondis, Nijmegen, The Netherlands, investigating new targets, particularly in cancer, that will lead to novel treatments. Jonathan is a PhD student in the lab of Marc Vooijs at Maastricht University, Maastricht, The Netherlands, investigating new cancer targets and testing possible new interventions with a focus on tumour hypoxia.


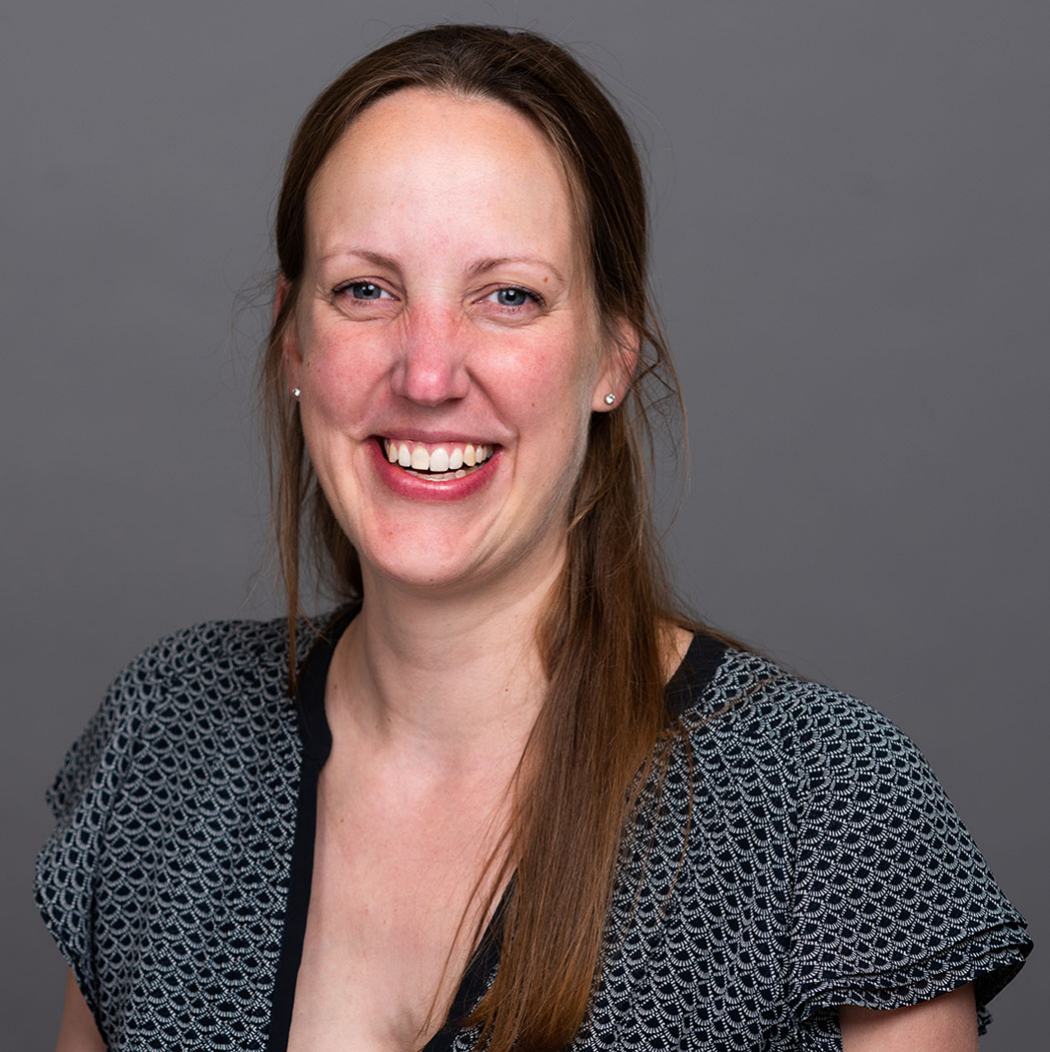


**Jenny Vermeer**


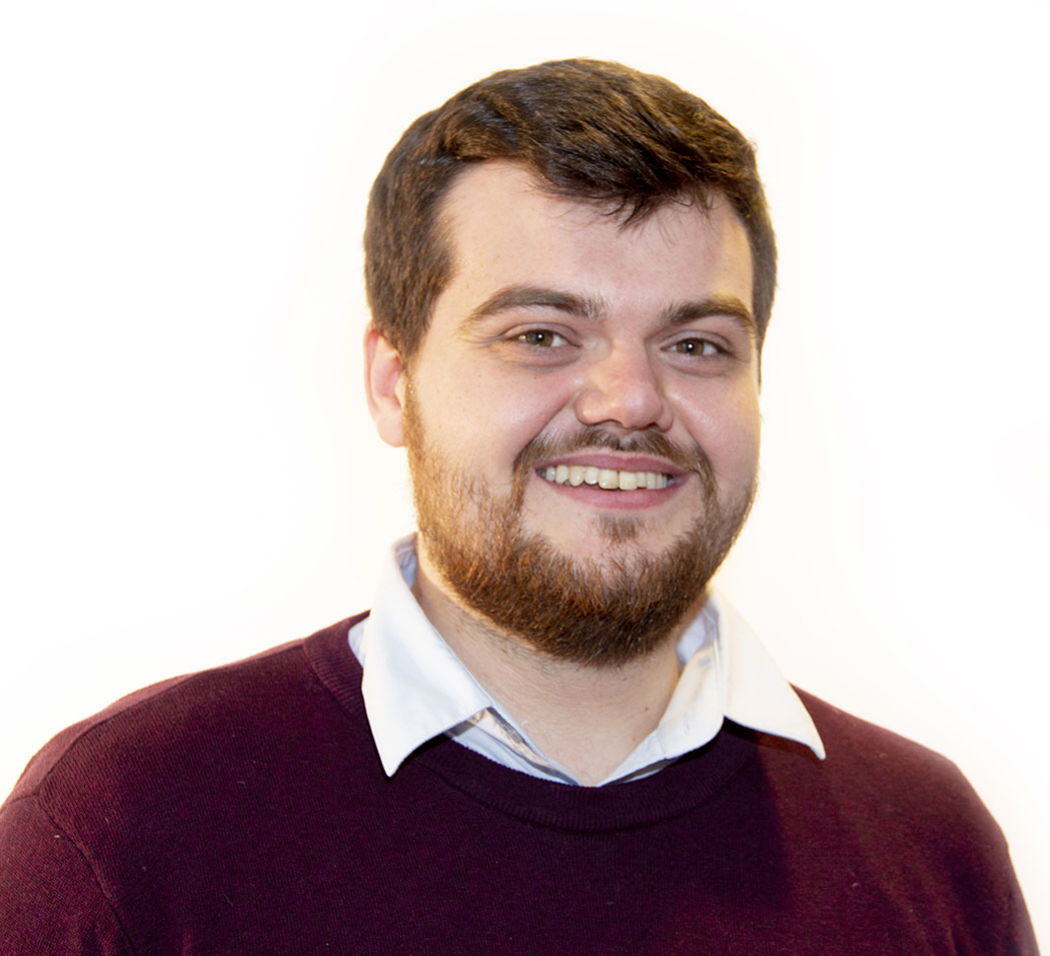


**Jonathan lent**

**How would you explain the main findings of your paper to non-scientific family and friends?**

**JV:** Cancer cells in a tumour grow uncontrollably. Due to their rapid expansion, it is difficult to provide enough oxygen for their growth. The state of low oxygen levels is called ‘hypoxia’ and it often makes tumours resistant to treatment. We visualised hypoxic cells in a mouse tumour with a microscope. This model makes it possible to study hypoxia in great detail and this knowledge can help to improve treatment strategies.

**JI:** Hypoxia has been known to increase malignant behaviour and treatment resistance, but clinical approaches to inhibit tumour hypoxia have failed. We have created a tool to study the behaviour of cells that have experienced hypoxia in real time, which will be useful in understanding how they contribute to treatment resistance.

**What are the potential implications of these results for your field of research?**

**JV:** The model we developed opens up a great opportunity to study hypoxic cells in more detail and gain knowledge about their fate and response to treatment.

**JI:** This model provides a tool for the community to use to study hypoxic cells from normal and diseased tissues at a single-cell level to help us understand the complex changes that hypoxia can cause. We believe this tool applied to cancer hypoxia will be key to identifying optimal therapeutic windows for new hypoxia modification strategies.

**What are the main advantages and drawbacks of the model system you have used as it relates to the disease you are investigating?**

**JV:** The main advantage of this model is that the long-term fate of hypoxic cells can be monitored. The model needs more optimisation to make it possible to simultaneously study currently hypoxic cells.

**JI:** The ability to study post-hypoxic cells live and at single-cell resolution rather than only at a fixed point in time. The study of cells that are currently under very low oxygen conditions requires more optimisation but with newer fluorescent proteins that do not require oxygen this can be overcome and can easily be introduced into the model.

**What has surprised you the most while conducting your research?**

**JV:** Hypoxia is very difficult to study due to its dynamics. The constantly changing microenvironment in a tumour makes hypoxia a very interesting and challenging subject.

**JI:** The range of different effects that hypoxia has is huge, and how all of these effects interact make it both interesting and often challenging to study.

**Describe what you think is the most significant challenge impacting your research at this time and how will this be addressed over the next 10 years?**

**JI:** The differences between models in the lab and patients often make it difficult to know if treatments will work in patients, but with the development of new models such as organoids derived from patient material, hopefully some of these problems can be overcome.

**Figure:**
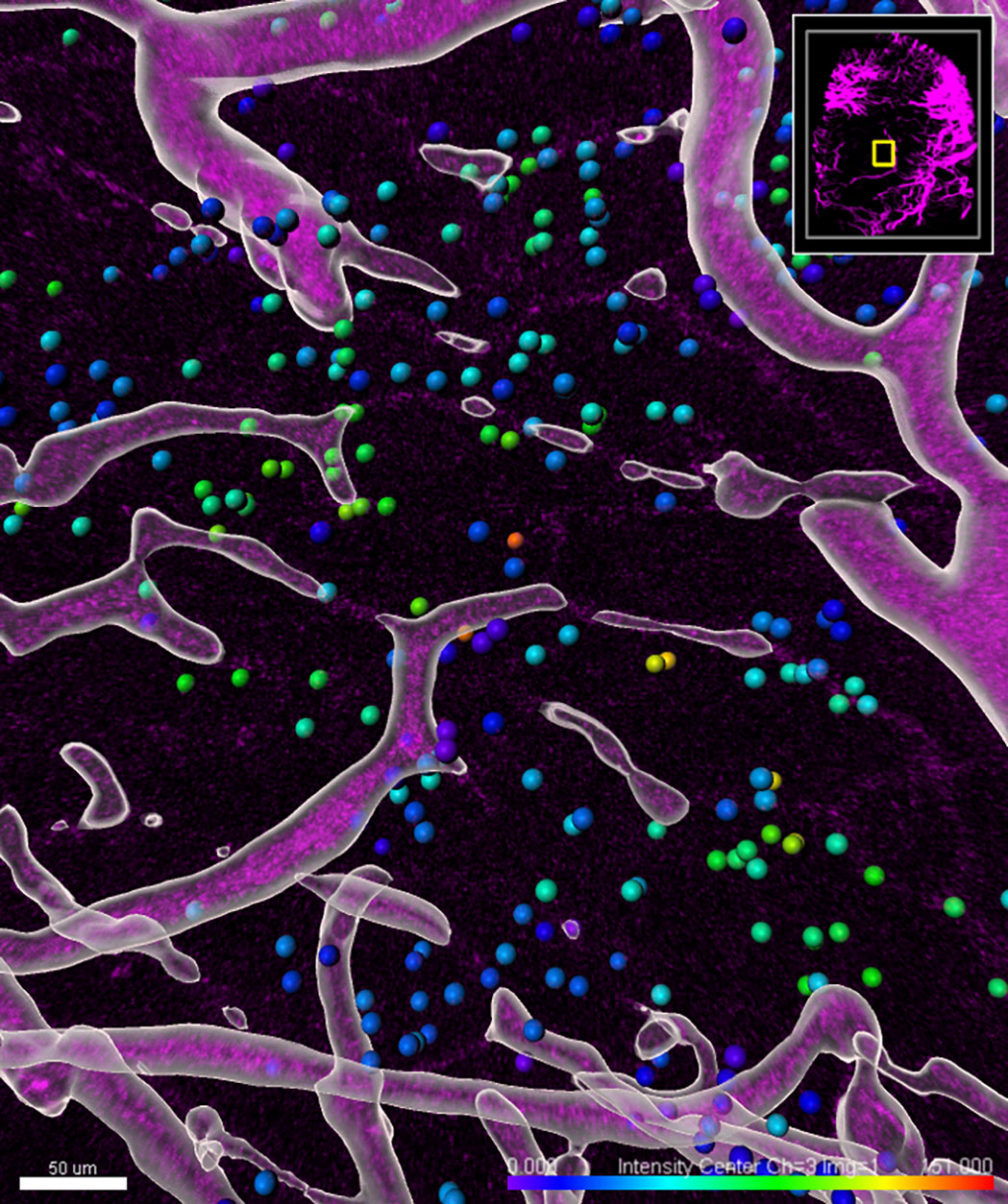
**Tumour vasculature (purple) and post-hypoxic cells (dots) imaged by intravital microscopy and masked with Imaris.**

“You yourself are responsible for your next step, it is never too early to think about it, and it should always be a high priority.”

**What changes do you think could improve the professional lives of early-career scientists?**

**JV:** I believe you should always be aware of your options and take matters into your own hands. You yourself are responsible for your next step, it is never too early to think about it, and it should always be a high priority.

**JI:** I agree with Jenny, but as well as that I think that the balancing of work with your social life is important, it is a lot easier to be motivated day to day if you have that balance, and that can often show through in your work.

**What's next for you?**

**JV:** After we submitted the manuscript, I moved from Oxford back to The Netherlands, where I am now working in early drug discovery research at the biopharmaceutical company Byondis.

**JI:** I am in the final months of my PhD and need to submit and defend. After that I will be looking for a postdoc position to carry on in research.

**Do you have any other remarks regarding this study?**

**JV:** This collaboration with Jon and Marc and the team has been a great journey. We started by occasionally meeting over Skype to discuss our plans and results. Along the way and when we were getting closer to writing the manuscript, we ‘met’ more often. Their excitement and optimism about the results have always been a great motivation.

**JI:** The collaboration with Jenny and Ruth has been very rewarding and a new experience for me with it mainly taking place over Skype and email rather than face to face. The mutual optimism and drive made it very enjoyable.
